# In Vitro Antifungal Drug Susceptibility of Feline *Sporothrix schenckii* Complex Isolates, Thailand, 2023–2025

**DOI:** 10.3201/eid3206.260135

**Published:** 2026-06

**Authors:** Chompoonek Yurayart, Kanokporn Yingchanakiat, Amonrat Thongbai, Orawan Limsivilai, Sathidpak Nantasanti Assawarachan, Tassanee Jaroensong, Panpicha Sattasathuchana

**Affiliations:** Faculty of Veterinary Medicine, Kasetsart University, Bangkok, Thailand

**Keywords:** *Sporothrix schenckii*, fungal infections, fungi, zoonoses, antimicrobial resistance, cats, felines, sporotrichosis, antifungal drug susceptibility, Thailand

## Abstract

Feline sporotrichosis is a public health concern because it is a zoonotic illness and has a prolonged treatment time. We report laboratory-confirmed cases of feline *Sporothrix schenckii* complex infection submitted to a laboratory in Thailand during 2023–2025 and evaluate their in vitro antifungal drug susceptibility profiles for amphotericin B, itraconazole, posaconazole, voriconazole, ketoconazole, fluconazole, and flucytosine. A total of 1,178 *S. schenckii* complex isolates were identified. Overall, 368 isolates did not show reduced susceptibility. We observed single-drug reduced susceptibility to amphotericin B (60 isolates), posaconazole (41 isolates), voriconazole (9 isolates), and terbinafine (4 isolates). We observed a reduced susceptibility to itraconazole in 687 isolates: 321 single-drug, 270 co-reduced, and 96 multidrug-reduced isolates. An increasing number of feline sporotrichosis cases and escalating reduced susceptibility to itraconazole underscore the need for continued surveillance and susceptibility testing to support management in complex cases.

*Sporothrix schenckii* complex is a group of genetically related fungi within the genus *Sporothrix*, which includes *S. schenckii* sensu stricto, *S. brasiliensis*, *S. globosa*, *S. mexicana*, *S. luriei*, and *S. albicans* (*1*). *S. schenckii* are thermal dimorphic pathogens that live and grow in mold form in the environment (*1*,*2*). Humans and animals become infected through the implantation of spores from contaminated materials into subcutaneous tissue through cuts and scratches; the fungus then converts to yeast form within the host tissue (*2*). *S. schenckii* complex causes sporotrichosis in humans and animals; cats are the most susceptible species. The geographic distribution, pathogenicity, clinical manifestations, virulence, and antifungal drug susceptibility differ among species. *S. brasiliensis* is the most virulent and highly prevalent, causing animal sporotrichosis and zoonotic infections in Brazil and other South American countries. *S. globosa* is an emerging human pathogen in China and India, and human infection occurs through environmental transmission (*3*–*5*). *S. schenckii* s.s. has occasionally been reported as causing environmentally acquired infection globally. However, a specific clade of *S. schenckii* s.s. has recently emerged among cats in Thailand, and reports of cat-to-cat and cat-to-human transmission are increasing (*6*–*8*). Cats with sporotrichosis exhibit multiple ulcerative mucosal and cutaneous lesions. Infected cats can shed infectious yeast organisms through saliva, nasal secretions, wound exudates, and scratches or bites to humans, cats, and other species (*9*). Cats serve as a major reservoir and highly efficient transmitter of sporotrichosis (*9*,*10*).

The effective treatment of infected cats with antifungal drugs is essential for controlling feline sporotrichosis. Performing antifungal drug susceptibility testing and analyzing the MIC values of endemic strains are necessary for monitoring antifungal drug susceptibility patterns. Clinical decisions are typically made on the basis of established clinical breakpoints; however, the clinical breakpoints for *Sporothrix* spp. are currently unavailable (*11*). Therefore, characterizing wild-type (WT) or non–wild-type (non-WT) isolates on the basis of the epidemiologic cutoff values (ECVs) is useful for surveillance of emerging antifungal drugs resistance (*12*). WT isolates have no resistance mechanisms and typically exhibit expected antifungal drugs susceptibility profiles, whereas non-WT strains have shown evidence of acquired or mutational changes, which reduce their susceptibility to antifungal drugs and are associated with higher MICs. Sporotrichosis treatment requires the use of antifungal drugs for several months, which might lead to antifungal drug resistance in clinical cases. Itraconazole is the antifungal drug of choice and the first-line treatment for sporotrichosis (*13*,*14*). Other antifungal drugs, including azoles (i.e., fluconazole, ketoconazole, posaconazole, and voriconazole), polyene (i.e., amphotericin B), pyrimidine analog (i.e., flucytosine), and allylamine (i.e., terbinafine), have also been used as combination therapies for human and feline sporotrichosis and other dimorphic fungal infections to shorten the treatment duration and improve clinical outcomes (*13*,*15*–*17*). Data on antifungal drug susceptibility from feline *S. schenckii* s.s. isolates in Thailand (2018–2021) reported in 2022 and 2023 (*6*,*18*) showed that prolonged treatment and frequent combination therapy might alter susceptibility patterns and lead to more complex resistance. We report laboratory-confirmed cases of feline sporotrichosis in Thailand from 2023–2025; characterize the in vitro antifungal drugs susceptibility profiles of *S. schenckii* complex isolates from cats in Thailand to amphotericin B, itraconazole, posaconazole, voriconazole, terbinafine, ketoconazole, fluconazole, and flucytosine; define the WT population of those isolates from 2023–2025; and determine the cross-reduced susceptibility patterns of itraconazole and other antifungal drugs.

## Materials and Methods

### Fungal Isolates and Data Collection

All the sample collection and handling procedures were approved by the Institutional Animal Care and Use Committee at Kasetsart University (approval no. ACKU68-VET-085). Clinical samples from cats suspected of feline sporotrichosis, submitted during 2023–2025 from veterinary clinics nationwide to the Department of Microbiology and Immunology, Faculty of Veterinary Medicine, Kasetsart University, Bangkok, were included.

We inoculated wound swab samples collected from cats with ulcerated cutaneous lesions in Sabouraud dextrose agar (SDA) at 30°C and brain–heart infusion agar containing gentamicin (50 mg/L; General Drugs House, https://generaldrugshouse.com) at 35°C for 5–14 days. We identified the causative agent as *S. schenckii* complex on the basis of its thermal dimorphic characteristics: a yeast form on brain–heart infusion agar at 35°C and a mold form as a hyaline septate fungus with a flower-like arrangement of the rosette and sessile conidia on SDA at 30°C. We subcultured and stored the isolates in sterile water for further antifungal drug susceptibility testing. For each cat, we obtained data on the age, sex, breed, body weight, and retrovirus-infected status (feline leukemia virus [FeLV] and feline immunodeficiency virus [FIV]) from hospital database and sample submission.

### Antifungal Drug Susceptibility Testing

We tested the *S. schenckii* complex isolates for their susceptibility to 8 antifungal drugs: amphotericin B, itraconazole, posaconazole, voriconazole, ketoconazole, terbinafine, fluconazole, and flucytosine. We performed antifungal drug susceptibility testing by using the broth microdilution method (BMD) according to Clinical and Laboratory Standards Institute (CLSI) for filamentous fungi guidelines (CLSI M38-A3) (*19*,*20*). The final concentrations of all the drugs assigned for testing were 0.06–32 μg/mL for amphotericin B, itraconazole, and posaconazole; 0.12–64 μg/mL for voriconazole; 0.015–8 μg/mL for terbinafine and ketoconazole; and 0.5–256 μg/mL for fluconazole and flucytosine. We sourced the antifungal drugs from MilliporeSigma (Sigma Aldrich, https://www.sigmaaldrich.com). We performed antifungal drug susceptibility testing against the mold form of the *S. schenckii* complex isolates as described previously (*6*). We dissolved the antifungal drugs according to CLSI M38-A3 (*20*) and diluted in Roswell Park Memorial Institute 1640 medium (Invitrogen, https://www.invitrogen.com) buffered to a pH of 7.0 with 0.65 M of 3-(N-morpholino)-propanesulfonic acid (MOPS) buffer (Millipore Sigma) with L-glutamine and without bicarbonate to achieve 2× the final concentration of each drug. We then loaded the solution into 96-well U-shaped microplates. We harvested a conidia suspension from the 3-day-old cultures growing on SDA at 30°C and adjusted the cell density to an absorbance of 0.09–0.11 at 530 nm and diluted at 1:50 in an RPMI 1640 culture medium to obtain the final cell concentration necessary for testing. We incubated the inoculated microplate at 35°C for 72 hours and determined the MIC visually. We included *Candida parapsilosis* ATCC 22019 to verify the accuracy of antifungal drug concentrations and potency in BMD plates but not as a quality control strain for filamentous fungi susceptibility testing.

### Antifungal Drug Susceptibility Analysis and Interpretation

To enable the parallel evaluation of the ECVs with those previously reported (*21*), we analyzed the MIC values of the isolates from Thailand. We calculated the MICs required to inhibit 50% (MIC_50_), 90% (MIC_90_), 95% (MIC_95_), 97.5% (MIC_97.5_), and 99% (MIC_99_) of the tested isolates. In addition, we determined the statistic mode by the highest frequency of MIC value for each antifungal drug. We identified the non-WT isolates as *S. schenckii* isolates with MIC values higher than the ECVs previously reported for amphotericin B (4 μg/mL), itraconazole (2 μg/mL), posaconazole (2 μg/mL), and voriconazole (64 μg/mL). The ECV for terbinafine has not yet been established; therefore, we defined a study-specific cutoff for terbinafine by statistical cutoff value at MIC_97.5_, according to CLSI M57 guidelines (*22*).

We used JMP Pro version 10 (SAS Institute, https://www.sas.com), GraphPad Prism version 9.0 (GraphPad, https://www.graphpad.com), and Stata version 14.2 (StataCorp, LLC, https://www.stata.com) for statistical analyses. We used Fisher exact test to assess the associations between the retroviral infections and non-WT populations. We also determined the associations between the non-WT isolates among the antifungal drugs. We considered results statistically significant when p<0.05.

## Results

We identified 1,178 *S. schenckii* complex isolates over the 3-year sampling period. Of those, in 2023 we recovered 225 isolates, in 2024 we recovered 383 isolates, and in 2025 we recovered 570 isolates. The demographic characteristics of cats with *S. schenckii* complex (Table 1) included mean +SD age of 4.0 +3.3 years and mean +SD bodyweight of 3.83 +1.3 kg. Most of the cats were male (60.7%) and domestic shorthairs (91.6%). Retroviral testing was performed on 253 cats, of which 28.1% were positive for FeLV or FIV.

**Table 1:** Demographic characteristics of 1,178 cats with sporotrichosis in Thailand, 2023–2025*

Parameter	Cats with sporotrichosis
Total no.	1,178
Age, y	
Median (range)	3.0 (0.1–21.3)
Mean +SD	4.0 +3.3
Sex	
Female	428 (36.3)
Male	715 (60.7)
Unknown	35 (3.0)
Breed	
American shorthair	2 (0.2)
Bengal	1 (0.1)
British shorthair	4 (0.3)
Domestic shorthair	1,079 (91.6)
Munchkin	2 (0.2)
Persian	27 (2.3)
Scottish fold	26 (2.2)
Unknown	36 (3.1)
Bodyweight, kg	
Median (range)	3.8 (0.5–9.4)
Mean +SD	3.83 + 1.3
Retrovirus infection test, total no.	253
FeLV or FIV negative	182 (71.9)
FeLV or FIV positive	71 (28.1)

*Values are no. (%) except as indicated. FeLV, feline leukemia virus; FIV, feline immunodeficiency virus

We determined the MIC_50_​, MIC_90_, MIC_95_​, MIC_97.5_​, MIC_99_​ and mode MIC values for all tested antifungal drugs (Table 2). Amphotericin B demonstrated MIC values ranging from 4 μg/mL (MIC_50_) to 16 μg/mL (MIC_99_​). Itraconazole and posaconazole exhibited high MIC values, with MIC_50_ values of 8 μg/mL (itraconazole) and 1 μg/mL (posaconazole), and MIC_90_​–MIC_99_​ exceeding 32 μg/mL. Voriconazole exhibited MIC values from 32 μg/mL (MIC_50_) to >64 μg/mL (MIC_99_​), whereas the MIC values for terbinafine ranged from 1 μg/mL (MIC_50_​) to 8 μg/mL (MIC_99_​). The MIC values were between 0.5–4 μg/mL for ketoconazole. Fluconazole and flucytosine had consistently high MIC values, with MIC_50_ ​to MIC_99_​ >64 μg/mL. The study-specific cutoff for terbinafine was assigned as 4 μg/mL according to MIC_97.5_.

**Table 2:** Antifungal drug susceptibility profiles of 8 antifungal drugs against 1,178 *Sporothrix schenckii* complex isolates in Thailand, 2023–2025*

Antifungal drug	MICs, μg/mL						
	Range	Mode	MIC_50_	MIC_90_	MIC_95_	MIC_97.5_	MIC_99_
Amphotericin B	0.06–>32	4 (WT)	4 (WT)	8 (non-WT)	8 (non-WT)	8 (non-WT)	16 (non-WT)
Itraconazole	0.06–>32	>32 (non-WT)	8 (non-WT)	>32 (non-WT)	>32 (non-WT)	>32 (non-WT)	>32 (non-WT)
Posaconazole	0.06–>32	1 (WT)	1 (WT)	>32 (non-WT)	>32 (non-WT)	>32 (non-WT)	>32 (non-WT)
Voriconazole	0.5–>64	64 (WT)	32 (WT)	64 (WT)	64 (WT)	>64 (non-WT)	>64 (non-WT)
Terbinafine	0.015–>8	1 (WT)	1 (WT)	2 (WT)	4 (WT)	4 (WT)	8 (non-WT)
Ketoconazole	0.015–>8	1	0.5	2	2	2	4
Fluconazole	1–>256	>256	>256	>256	>256	>256	>256
Flucytosine	0.5–>256	64	64	>256	>256	>256	>256

*Mode is the MIC that occurs most frequently. MIC_50_, MIC_90_, MIC_95_, MIC_97.5_, and MIC_99_ values are reported as the minimum concentrations of antifungal drugs required to inhibit 50%, 90%, 95%, 97.5%, and 99% of the growth of *S. schenckii* complex isolates. Susceptibility phenotypes were characterized on the basis of available epidemiologic cutoff values (*21*) and study-specific cutoff value (for terbinafine). Non-WT, non-wild-type; WT, wild-type.

We observed a predominance of WT isolate susceptibility toward amphotericin B (79.0%), posaconazole (73.9%), voriconazole (97.3%), and terbinafine (98.2%). In contrast, itraconazole revealed a predominance of non-WT isolate susceptibility (58.3%). The distribution of MIC values for amphotericin B, itraconazole, posaconazole, voriconazole, and terbinafine and MICs for antifungal drugs that do not have designated ECVs for *S. schenckii* complex are provided (Table 3).

**Table 3:** MIC distribution and susceptibility phenotypes for 8 antifungal drugs against 1,178 *Sporothrix schenckii* complex isolates in Thailand, 2023–2025*

Antifungal drugs	Susceptibility phenotypes			Distribution of MICs, μg/mL																
	WT	Non-WT		0.015	0.03	0.06	0.12	0.25	0.5	1	2	4	8	16	32	64	128	256	>256	
Amphotericin B	931 (79.0)	247 (21.0)		NA	NA	3	6	27	70	48	187	**590**	234	9	2	2‡	NA	NA	NA	
Itraconazole	491 (41.7)	687 (58.3)		NA	NA	3	3	38	86	159	202	104	70	40	24	**449**‡	NA	NA	NA	
Posaconazole	870 (73.9)	308 (26.2)		NA	NA	1	3	69	165	**388**	244	45	169	12	9	73‡	NA	NA	NA	
Voriconazole	936 (97.3)	26 (2.7)		NA	NA		0	0	3	9	18	61	193	171	236	**245**	26‡	NA	NA	
Terbinafine§	1,157 (98.2)	21 (1.8)		2	2	11	42	198	330	**333**	168	71	14	7‡	NA	NA	NA	NA	NA	
Ketoconazole	ND	ND		4	3	18	71	177	304	**379**	187	31	3	1‡	NA	NA	NA	NA	NA	
Fluconazole	ND	ND		NA	NA	NA	NA	NA	0	1	0	1	4	1	2	6	48	131	**984**‡	
Flucytosine	ND	ND		NA	NA	NA	NA	NA	4	0	5	29	70	85	209	**246**	197	114	3‡	

*Values are no. (%) except as indicated. Bold indicates highest number in each row (mode). Gray shading indicates non-WT. NA, not available because it was not tested; ND, not determined because of the lack of an epidemiologic cutoff value and the drug not being commonly used; non-WT, non-wild-type; WT, wild-type. †The MIC is compared with the *S. schenckii* epidemiologic cutoff value (*21*). ‡The MICs were greater than the highest tested concentration (>X μg/mL). X was replaced with the actual maximum concentration of each drug (e.g., >32 μg/mL). §Terbinafine susceptibility phenotypes are characterized by a study-specific cutoff value based on data in the study, that is, the MIC of antifungal drugs required to inhibit 97.5% of the growth of *S. schenckii* complex isolates (4 μg/mL).

We did not find an association between retroviral infections and non-WT isolates for amphotericin B (p = 0.2367), itraconazole (p = 0.2569), posaconazole (p = 0.2367), voriconazole (p>0.9999), and terbinafine (p>0.9999). p. 928 clarification left column: We observed significant associations between retroviral infections and non-WT isolates for itraconazole isolates and non-WT isolates for amphotericin B (p<0.0001), posaconazole (p<0.0001), and terbinafine (p = 0.0428). The non-WT posaconazole isolates for posaconazole were also significantly associated with the non-WT isolates for amphotericin B (p<0.0001) and terbinafine (p = 0.0005).

We did not observe reduced susceptibility to any of the tested antifungal drugs in 368 isolates (31.2%). We observed single-drug reduced susceptibility to amphotericin B in 60 isolates, to posaconazole in 41 isolates, to voriconazole in 9 isolates, and to terbinafine in 4 isolates. We also observed cross-reduced susceptibility in 8 isolates between amphotericin B and posaconazole, and 1 isolate demonstrated cross-reduced susceptibility between amphotericin B and voriconazole. For itraconazole, 491 isolates showed no reduced susceptibility pattern, whereas 687 isolates exhibited reduced susceptibility. We observed single-drug reduced susceptibility to itraconazole in 321 isolates. We identified co-reduced susceptibility with itraconazole in 270 isolates and observed multidrug reduced susceptibility to itraconazole in 96 isolates (Table 4).

**Table 4:** Distribution of non-WT phenotypes and antifungal drugs with co-reduced susceptibility patterns based on itraconazole susceptibility among feline *Sporothrix schenckii* complex isolates in Thailand, 2023–2025 (n = 1,178)

Itraconazole susceptibility phenotype	No. drugs, non-WT	Itraconazole reduced susceptibility patterns	Frequency	Overall
WT	0	None	491 (41.7)	491 (41.7)
Non-WT	1	Itraconazole	321 (27.2)	321 (27.2)
	2	Itraconazole + posaconazole	165 (14.0)	270 (22.9)
		Itraconazole + amphotericin B	94 (8.0)	
		Itraconazole + voriconazole	7 (0.6)	
		Itraconazole + terbinafine	4 (0.3)	
	3	Itraconazole + posaconazole + amphotericin B	74 (6.3)	88 (7.5)
		Itraconazole + posaconazole + terbinafine	7 (0.6)	
		Itraconazole + posaconazole + voriconazole	5 (0.4)	
		Itraconazole + voriconazole + amphotericin B	2 (0.2)	
	4	Itraconazole + posaconazole + amphotericin B + terbinafine	6 (0.5)	8 (0.7)
		Itraconazole + posaconazole + amphotericin B + voriconazole	2 (0.2)	

*Values are no. (%) except as indicated. Non-WT is defined by previously reported epidemiologic cutoff values (*21*) and study-specific cutoff value (for terbinafine). Non-WT, non-wild-type; WT, wild-type.

## Discussion

We phenotypically identified 1,178 feline *S. schenckii* complex isolates by fungal culture and determined them to have in vitro antifungal drug susceptibilities. Only 31.2% of all the isolates were susceptible to all the tested drugs. Terbinafine, voriconazole, amphotericin B, and posaconazole exhibited high efficacy, whereas itraconazole had limited efficacy. No association was apparent between the retroviral infections and non-WT isolates for amphotericin B, itraconazole, posaconazole, voriconazole, and terbinafine. Various patterns of in vitro reduced susceptibility to antifungal drugs were characterized in this study, including single-drug reduced, co-reduced, and multidrug reduced susceptibility.

Samples were obtained from client-owned cats taken to veterinary clinics and with suspected feline sporotrichosis. Specimens were collected by attending veterinarians for routine diagnostic procedures. During 2023–2025, the number of cats diagnosed with sporotrichosis in Thailand increased steadily: 225 cases in 2023, increasing to 383 cases in 2024 and 570 cases in 2025. Reported cases represent laboratory-confirmed infections. Not all infected cats might have undergone confirmatory testing. As a result, the number of cases reported in this study likely underestimates the true number of feline sporotrichosis infections in Thailand. Although samples were submitted from multiple veterinary clinics across the country, geographic distribution was not analyzed in this study. Further epidemiologic studies are warranted to better define the incidence and prevalence of feline sporotrichosis in Thailand.

In this study, most of the cats were young adult, male, and domestic shorthair cats that were seronegative for FeLV and FIV. Those findings align with previously reported cases of 38 cats with sporotrichosis in Thailand during 2020–2022 (*6*). Young adult male cats exhibit roaming behaviors, which increases their exposure to pathogens. No specific breed has shown a predisposition for *S. schenckii* complex, and all cat breeds can be infected with this pathogen (*6*). The predominance of domestic shorthair cats likely reflects their high ownership in Thailand (*23*). Although retrovirus infections impair immune function by reducing the CD4+/CD8+ lymphocyte ratio (*24*), we found no association between the isolates from cats with seropositive retrovirus and those with non-WT phenotypes. The occurrence of non-WT *S. schenckii* complex isolates might be associated with virulent strains or prior antifungal drug exposure, rather than the underlying immune status of the host.

We used the CLSI M38-A3 BMD method as the reference standard for the surveillance of drug susceptibility in *Sporothrix* spp.; however, its complexity and the need for microbiological expertise limit its use in routine clinical settings. Commercially available antifungal drugs susceptibility testing platforms have limitations. The Vitek 2 (bioMérieux, https://www.biomerieux.com) automated system is widely used for antifungal drug susceptibility testing of *Candida* spp. and *Cryptococcus* spp. (*25*). The Vitek 2 has limited applicability for *Sporothrix* spp. because of the dimorphic nature and slow growth kinetics of the species. Likewise, discordance between Sensititer YeastOne (Thermo Fisher Scientific, https://www.thermofisher.com) and the CLSI reference method has been reported (*26*). The Sensititer YeastOne panel lacks key antifungal drugs, including terbinafine, which is the second-line treatment recommended drug for sporotrichosis (*26*,*27*). Using the CLSI M38-A reference method is essential for establishing an accurate baseline of antifungal drug susceptibility, particularly because of the increasing number of sporotrichosis cases in Thailand. The future development of user-friendly commercial antifungal drug susceptibility testing platforms for *Sporothrix* spp. would support the routine diagnosis and clinical management of sporotrichosis among physicians and veterinarians.

In this study, we applied established ECVs for *S. schenckii* complex to distinguish between the WT and non-WT MIC distributions for amphotericin B, itraconazole, posaconazole, and voriconazole (*21*). A study-specific cutoff at MIC_97.5_ was used to characterize the susceptibility profile of the feline *S. schenckii* complex isolates from Thailand to terbinafine. Most of the testing isolates were obtained from clinical cases that were unresponsive to prior antifungal drug therapy or were specifically submitted for MIC testing to guide veterinarians’ clinical decision-making. The high MIC values observed might reflect a subset of reduced susceptibility or clinically challenging strains rather than the baseline susceptibility of feline *S. schenckii* complex isolates from Thailand.

Itraconazole demonstrated a remarkably reduced efficacy compared with previous data from an earlier outbreak in Thailand (*6*,*8*). In this study, most of the isolates were classified as non-WT to itraconazole by MIC_50_ and exhibited reduced susceptibility (MIC_90_ >32 μg/mL), which contrasts with previous findings in which most of the isolates were WT with MIC_50_ at 0.5 μg/mL and non-WT with MIC_90_ at 4 μg/mL (*6*). The reduced susceptibility to itraconazole phenotypes we observed were reflected in the 16-fold increase in MIC_50_ and >8-fold increase in MIC_90_; a >4-fold increase in these values is indicative of acquired reduced susceptibility in non-WT strains (*12*,*28*,*29*). Our findings align with previously reported MICs from feline and human isolates from Thailand that exhibited reduced susceptibility to itraconazole and identified non-WT isolates (*7*,*10*,*18*). Furthermore, among the itraconazole non-WT isolates, a high rate (31.1%) of cross-reduced susceptibility and multidrug reduced susceptibility was detected, with prominent reduced susceptibility to itraconazole/posaconazole and itraconazole/amphotericin B and the multidrug combination of itraconazole, posaconazole, and amphotericin B. Cross-reduced susceptibility between itraconazole and posaconazole is often documented because both azole drugs share a common molecular target and overlapping binding sites within the ergosterol biosynthesis pathway (*12*). Moreover, the widespread use of itraconazole in feline sporotrichosis in Thailand often involves prolonged or inconsistent dosing because of multiple factors, including drug cost, bioavailability, owner compliance, difficulties in administering medication to cats, zoonotic concerns, and adverse effects. Antifungal drug treatment is not subsidized, and financial constraints might affect treatment continuity. In addition, limited access to veterinary care for stray cats and the absence of structured control programs might contribute to ongoing transmission. Those challenges can lead to treatment interruption or incomplete therapy, which might promote reduced susceptibility and multidrug resistance (*12*,*30*–*33*).

A reduction in susceptibility to amphotericin B is a concern. We found that the MIC_50_ had shifted 8-fold (from 0.5 to 4 μg/mL) compared with previous data, in which non-WT strains were not detected (*6*). Amphotericin B is reserved for second-line therapy in severe disseminated sporotrichosis or in cases refractory to itraconazole (*31*,*34*). Most of the clinically challenging isolates in our study remained WT, with borderline susceptibility to amphotericin B. The use of amphotericin B as an empirical choice in those cases without prior antifungal drugs susceptibility testing would therefore have been precarious, because amphotericin B requires a minimum effective concentration of 4–8 µg/mL, which might risk dose-dependent nephrotoxicity (*35*).

Terbinafine is another second-line drug therapy used as a single treatment or in combination with itraconazole. Successful treatment with terbinafine remains controversial; some successful reports have been published in human and dog sporotrichosis cases, whereas the reported success rate in feline sporotrichosis caused by *S. brasiliensis* is low (*36*–*38*). In Thailand, this drug has been used frequently in cats when *S. schenckii* has failed to respond to itraconazole, and terbinafine has previously shown low MICs among *S. schenckii* isolates from Thailand and Malaysia that share closely related genotypes (*6*,*7*,*27*). In our study, terbinafine was the most potent agent in vitro, with consistently low MICs (1.3-fold increase in MIC_50_ and no change in MIC_90_) compared with those in previous reports (*6*). Cross-drug and multidrug reduced susceptibility to terbinafine, itraconazole, and other drugs were extremely low, especially among the non-WT isolates in our study. Our findings align with those of previous studies suggesting the use of terbinafine as an alternative drug for treating sporotrichosis against itraconazole non-WT isolates, either as a monotherapy or combined with posaconazole, on the basis of in vitro antifungal drug susceptibility testing and an in vivo *Galleria mellonella* model of *S. schenckii* infection (*18*).

This study had a very large and diverse collection of isolates from Thailand for in vitro susceptibility testing. Despite the clinically challenging nature of those isolates, terbinafine MICs remained stable and less variable than those of the other drugs, which indicated that the feline *S. schenckii* complex isolates from Thailand had a suitable susceptibility profile to terbinafine. We found diverse distributions of terbinafine MICs in the populations of *S. schenckii* complex native feline non-genotyped isolates from Thailand. In comparing our mode terbinafine value (mode MIC = 1 μg/mL) to that of a large, diverse population study (mode MIC = 0.5 μg/mL) (*21*), the difference was only 2-fold and not significant for susceptibility testing. Because of the increasing need for terbinafine to treat feline sporotrichosis and the rising rate of zoonotic infections in Thailand, we proposed a study-specific cutoff of 4 µg/mL for terbinafine for *S. schenckii* species complex. That cutoff might help monitor reduced susceptibility and support effective antifungal drug use, particularly because of the reduced susceptibility to itraconazole among strains in Thailand. Furthermore, including strains from Thailand in MIC datasets for ECV determination would broaden global studies on the susceptibility profiles of zoonotic *S. schenckii*.

The first limitation of this study is lack of correlation between the MICs and clinical treatment outcomes and the absence of disease severity analysis for non-WT isolates. Second, molecular identification was not performed to confirm species within the *S*. *schenckii* complex. Therefore, interpretation of MIC values based on ECVs for *S. schenckii* should be approached with caution. Feline sporotrichosis in Thailand is predominantly caused by *S. schenckii* s.s., supporting the use of those ECVs as a reference in this study (*6*–*8*), though species-level identification remains limited. A recent study proposed updated ECVs for *Sporothrix* species, which have been approved by the CLSI subcommittee and are expected in a future supplement (A.R. dos Santos, et al. unpub. data, https://www.biorxiv.org/content/10.64898/2025.12.19.695392v1). Those developments emphasize the necessity of species-level identification and standardized criteria for antifungal drug susceptibility testing and the need for standardized protocols to ensure consistent interpretation across studies. Future studies incorporating molecular identification are warranted to validate species-level distribution and improve the accuracy of antifungal drugs susceptibility interpretation. We primarily provided descriptive and surveillance data on in vitro antifungal drug susceptibility patterns and evidence of cross-reduced susceptibility. However, treatment outcomes cannot yet be reliably predicted from susceptibility profiles.

In conclusion, we found an increasing number of confirmed feline sporotrichosis cases in Thailand, with 1,178 cases reported within a 3-year period, together with the occurrence of reduced itraconazole susceptibility. Those results suggest that reliance on empirical therapy alone might be insufficient in refractory cases, as reduced susceptibility patterns can vary across regions and over time. In vitro drug susceptibility testing should be performed for disease surveillance and in selected complex cases.
